# Mobile road weather sensor calibration by sensor fusion and linear mixed models

**DOI:** 10.1371/journal.pone.0211702

**Published:** 2019-02-07

**Authors:** Lauri Lovén, Virve Karsisto, Heikki Järvinen, Mikko J. Sillanpää, Teemu Leppänen, Ella Peltonen, Susanna Pirttikangas, Jukka Riekki

**Affiliations:** 1 Infotech Oulu, University of Oulu, Oulu, Finland; 2 Finnish Meteorological Institute, Helsinki, Finland; 3 University of Helsinki, Helsinki, Finland; Newcastle University, UNITED KINGDOM

## Abstract

Mobile, vehicle-installed road weather sensors are becoming ubiquitous. While mobile sensors are often capable of making observations on a high frequency, their reliability and accuracy may vary. Large-scale road weather observation and forecasting are still mostly based on stationary road weather stations (RWS). Though expensive, sparsely located and making observations on a relatively low frequency, RWS’ reliability and accuracy are well-known and accommodated for in the road weather forecasting models. Statistical analysis revealed that road weather conditions indeed have a great effect on how the observations of mobile and stationary road weather temperature sensors differ from each other. Consequently, we calibrated the observations of mobile sensors with a linear mixed model. The mixed model was fitted fusing ca. 20 000 pairs of mobile and RWS observations of the same location at the same time, following a rendezvous model of sensor calibration. The calibration nearly halved the MSE between the observations of the mobile and the RWS sensor types. Computationally very light, the calibration can be embedded directly in the sensors.

## Introduction

Mobile, vehicle-installed sensors and road weather station (RWS) networks can together provide denser and higher quality information than either alone. They can support optimization of maintenance operations, such as snow clearance and prevention of slipperiness, and generation of real-time warnings for road users. Accurate now-casting and forecasting are keys to safe and economic operations, especially in the northern latitudes where driving conditions can vary a lot in space and time, increasing risk for accidents.

Improved technologies and increased availability of mobile observations can drastically improve the coverage and quality of observations on roads. The amount of available mobile observations have recently considerably increased. During November 2016—March 2017, for instance, vehicles fitted with Teconer Oy’s optical sensors (RCM411 and RTS411) covered globally approximately 200 000 km of roads per month observing friction, surface water deposits, and road surface temperature [1].

There have been several studies about the usability of mobile observations. For example, mobile road condition monitoring was tested in Finland already in 1998—1999 [2]. Also Stern et al. [3] studied the reliability of mobile observations, while Koller et al. [4] examined the quality of the observations with the native sensors of a vehicle. In 2013, Finnish transport agency compared optical friction and temperature meters [5]. The study compared, among other things, Teconer’s optical road surface temperature sensor RTS411 with RWS measurements. RTS411 gave typically 1.2°C warmer values than the RWS. Such a large difference between observations calls for sensor calibration.

Sensor calibration unifies sensor data that one or multiple sensors collect for a particular application. Drifting, zero offset errors, or variations in the manufacturing process can cause even sensors by the same manufacturer to yield different readings in the same conditions, with systematic or random errors. Measurement errors are aggravated when the sensors, during operation or storage, are subjected to varying environmental conditions such as light, temperature, humidity, hysteresis or shock. In addition, the selected sensor technology may initially provide low signal to noise-ratio, which makes repeatable measurements challenging. Thus, re-calibration in the field may be needed after initial factory calibration. This problem is magnified when different types of the same sensor modality are used to measure the same physical phenomena. In such a case, multiple sensor readings may be based on measuring completely different, but related, parameters of the monitored phenomena.

Typically, single sensors are calibrated with some physical reference, e.g. gravity, that provides a direct mapping of measured values to standardized units. For linear sensors, simple calibration can be based on reference points. For non-linear sensors, calibration typically requires multi-point curve fitting methods. For example, air quality sensor applications [6] use highly accurate weather stations as reference points, providing the ground truth. However, the sensing range and frequency provided by such stations is limited and the distances between stations result in a low spatial resolution.

The coverage and resolution of sparse, stationary sensors can be improved with mobile devices with integrated sensors, e.g. vehicle-installed smartphones [7]. However, smartphone sensing devices suffer from noisy measurements due to low-cost sensor technology [6]. Further, inexperienced users can cause errors in the data, for example by installing the sensors incorrectly. Finally, manual calibration by users may introduce uncertainty. Automatic calibration methods are thus used to eliminate the cumbersome and error-prone manual calibration [8]. One such automatic calibration method is the rendezvous model [9–12]. In the rendezvous model, observations by two or more sensors, mobile or stationary, are collected when the sensors are co-located, i.e. at the same time in the same place. The mobile sensors are calibrated by comparing the rendezvous observations. In addition to ensuring the spatio-temporal identity of the observations, the locations of the static reference points need to be carefully considered [8].

Sensor fusion refers to merging information from two or more sensors, located spatially close to each other. Sensor fusion helps with problems related to low spatial resolution and unreliable users. It has previously been used for example for autonomous vehicle navigation, improving lane [13] and road potholes [14] recognition. Rendezvous model uses sensor fusion to unify data from different sensors.

This study proposes a sensor fusion based method to calibrate mobile road weather sensors. Specifically, road surface temperature sensors are calibrated with the rendezvous model. The model compares spatio-temporally co-located observations by sparse Vaisala RWS sensors with the dense but possibly less reliable mobile observations provided by vehicle-installed Teconer sensors RCM411 and RTS411. The aim is to first chart the statistical characteristics of the mobile observations when compared to the RWS observations, and then calibrate the mobile sensors to agree with the RWSs.

The paper is organized as follows: Section *Methods and Data* presents the methodology, data, and statistical models used in the study, Section *Results and discussion* presents the results and discusses the outcome, and finally Section *Conclusion* concludes the study.

## Methods and data

### Overview

This study presents a novel, sensor fusion based method to calibrate mobile surface temperature sensors to agree with RWSs. The methodology is summarized as follows:

1. This study uses RWS and mobile data, collected during winter seasons between 2014–2017.
2. Rendezvous, i.e. occasions where the mobile sensors have passed one of the RWSs, are identified. The data on these rendezvous comprises the full data set, while the remaining data is discarded.
3. Relationships between the mobile observations and the RWS measurements are analysed with statistical models. Two particular research questions are considered:
  - How are the mobile observations related to environmental conditions such as weather?
  - How can we calibrate the mobile sensors, i.e. improve the accuracy of the mobile observations when compared to the RWS observations?
  Statistical inference models are built to answer the first question. Statistical prediction models are built to answer the second question.

### Data

**Fig 1:**
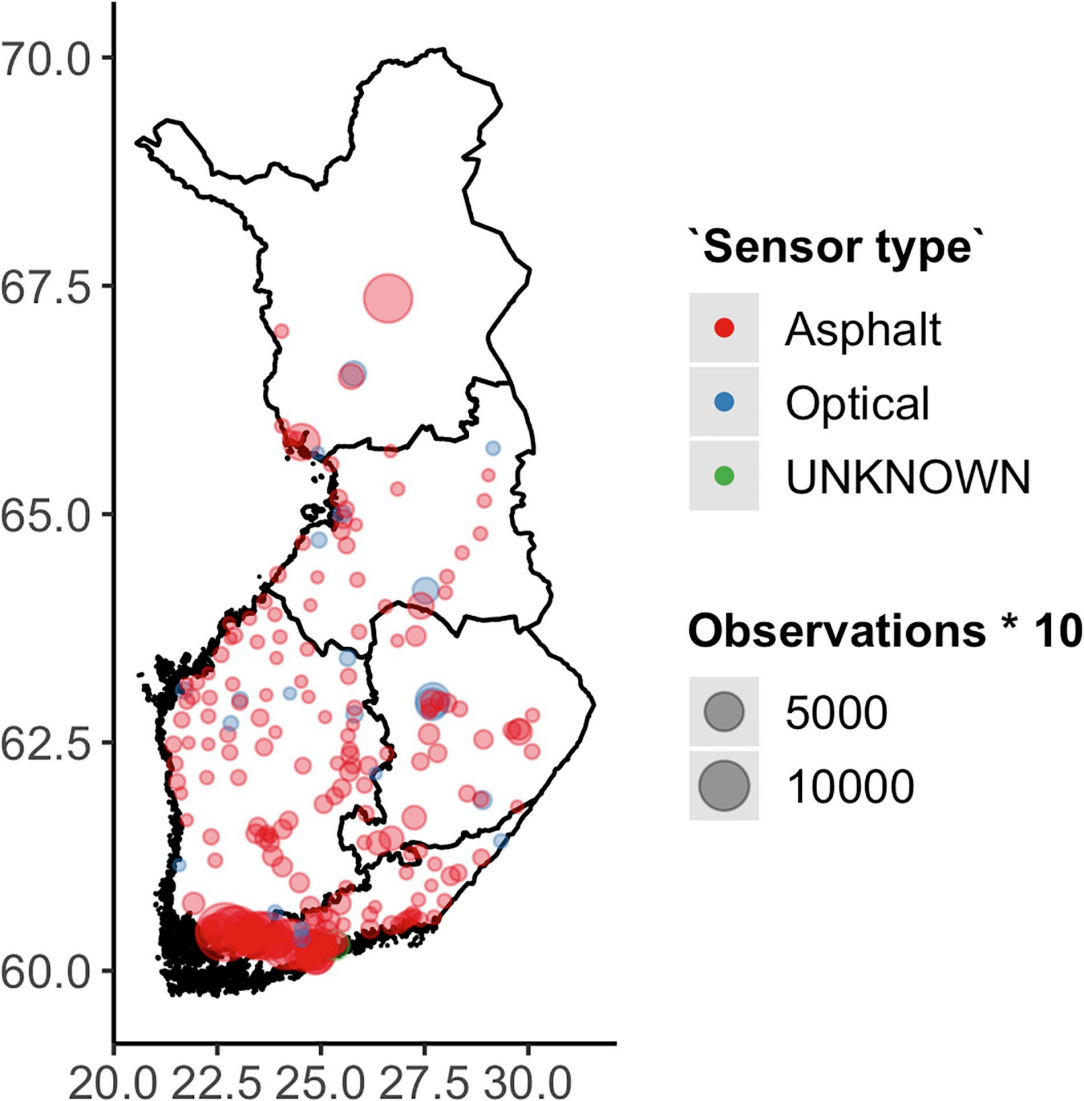
Spatial distribution of data. Coordinates are in latitude and longitude.

This study used road weather data collected by 251 stationary Vaisala RWSs, and 27 vehicles installed with Teconer RCM411/RTS411 sensors. Fig 1 shows the locations of the RWSs, mostly deployed at the major roads around southern and central Finland. The full data set contains 25944 observations. However, few RWSs were fitted with a full array of sensors, so in most cases some of the observed variables are missing.

The RWSs measure multiple weather variables, such as temperature and wind conditions. We used a representative subset of the available variables fitting to the road-condition analysis, which are listed in Table 1. First, road surface temperature is measured at most of the stations by an asphalt embedded DRS511 sensor [15]. Further, the DRS511 sensor also estimates overall road status. This can be, for example, “wet”, “icy”, or “snowy”. Observations whose road status is “error” are discarded.

**Table 1:** Data description.

Type	Source	Variable	Notes
Mobile	Teconer RTS411	Road surface temperature	Continuous (°C).
	Teconer RCM411	Road state	Categorical: Dry, Moist, Wet, Slush, Ice, or Snow/Frost.
	Teconer RCM411+GPS	Location	Continuous (lat,lon).
	Teconer RCM411	Sensor ID code	Categorical.
RWS	Vaisala DRS511/DST111	Road surface temperature	Continuous (°C).
	Vaisala DST111	Air temperature	Continuous (°C).
	Vaisala DRS511+DST111	Road state	Categorical: Dry, Moist, Wet, Wet&Salty, Frost, Snow, Ice, Moist&Salty, Slushy
	Vaisala DSC111	Road state	(as above)
	Vaisala DSC111	Water deposits	Continuous (mm).
	Vaisala DSC111	Ice deposits	Continuous (mm).
	Vaisala DSC111	Snow deposits	Continuous (mm).
	Vaisala DRS511/DST111	Sensor type	Categorical: Optical, Asphalt, or Unknown.
	Vaisala DRS511/DST111	Sensor ID code	Categorical. Individual id code for each vehicle installation.
Circumstantial		Year	Continuous.
		Season	Categorical: Fall (Sep–Nov), Winter (Dec–Mar), Spring (Apr).

Some RWSs have optical Vaisala DST111 sensors [16], measuring road surface temperature by the infrared radiation emitted by the road surface. DST111 measures also air temperature. However, optical sensors at Finnish RWSs are more commonly Vaisala DSC111s [17], which detect water, ice, and snow deposits on the road. The method is based on absorption wavelengths of water and ice. The device transmits infrared radiation with certain wavelengths, and the deposits are determined from the radiation backscattered from the surface [18]. For Vaisala DSC111, the resolution for water, ice and snow is reported as 0.01 mm [17]. DSC111 determines also the road status (“wet”, “icy” etc). (Observations with road status “error” were discarded.) DSC111 and DST111 sensors are typically installed on poles beside the road.

Some of the road weather stations contained multiple surface temperature sensors. These sensors were either optical or installed in the asphalt. In this study, only one such sensor for a station was used at a time. Thus, the Vaisala sensor identification code, telling apart the individual sensor devices, also differentiates between the RWSs.

The optical Teconer RCM411 road condition sensor, fitted on vehicles, has the same operational principle as the DSC111. It observes, among other things, overall road status (e.g. “wet”, “icy” etc.) [1]. An add-on on the RCM411, a Teconer RTS411 sensor measures road surface temperature based on infrared radiation similarly as Vaisala DST111.

RCM411+RTS411 device pairs can be connected to a nearby cell phone by a Bluetooth connection. The cell phone is used to obtain the location, direction and speed of the vehicle. These are also included in Table 1.

Teconer device identification codes differentiate between individual RCM411+RTS411 device pairs. However, as one device pair could have multiple vehicle installations over the observation period, and each installation could have a different calibration level, we modified the identification codes such that each installation had its own identification code.

The study data contains observations from three cold periods (September—April) between years 2014 and 2017. Firstly, we compare the mobile observations to the RWS locations, and then select those data points where a mobile sensor passed by an RWS within 50m or less. Each such occasion constitutes a rendezvous. As the mobile sensor observation rate is once per second, one rendezvous might comprise several observations. Thus, the numerical observations are averaged, and for the non-numerical observations (e.g. road status), we choose the one with the smallest distance between the RWS and the mobile sensors. However, the non-numerical observations stay constant during the vast majority of rendezvous’, indicating our choice of 50m radius is well chosen.

Rendezvous time is considered to be the middle time point between the first and the last observation within one pass. The RWS observation rate is once per 5-10 minutes, depending on the station. Thus, there is often a time discrepancy between an RWS observation and the mobile sensor pass time. The RWS observation with the smallest absolute time difference to the pass time is used in this study.

### Explorative analysis

**Fig 2:**
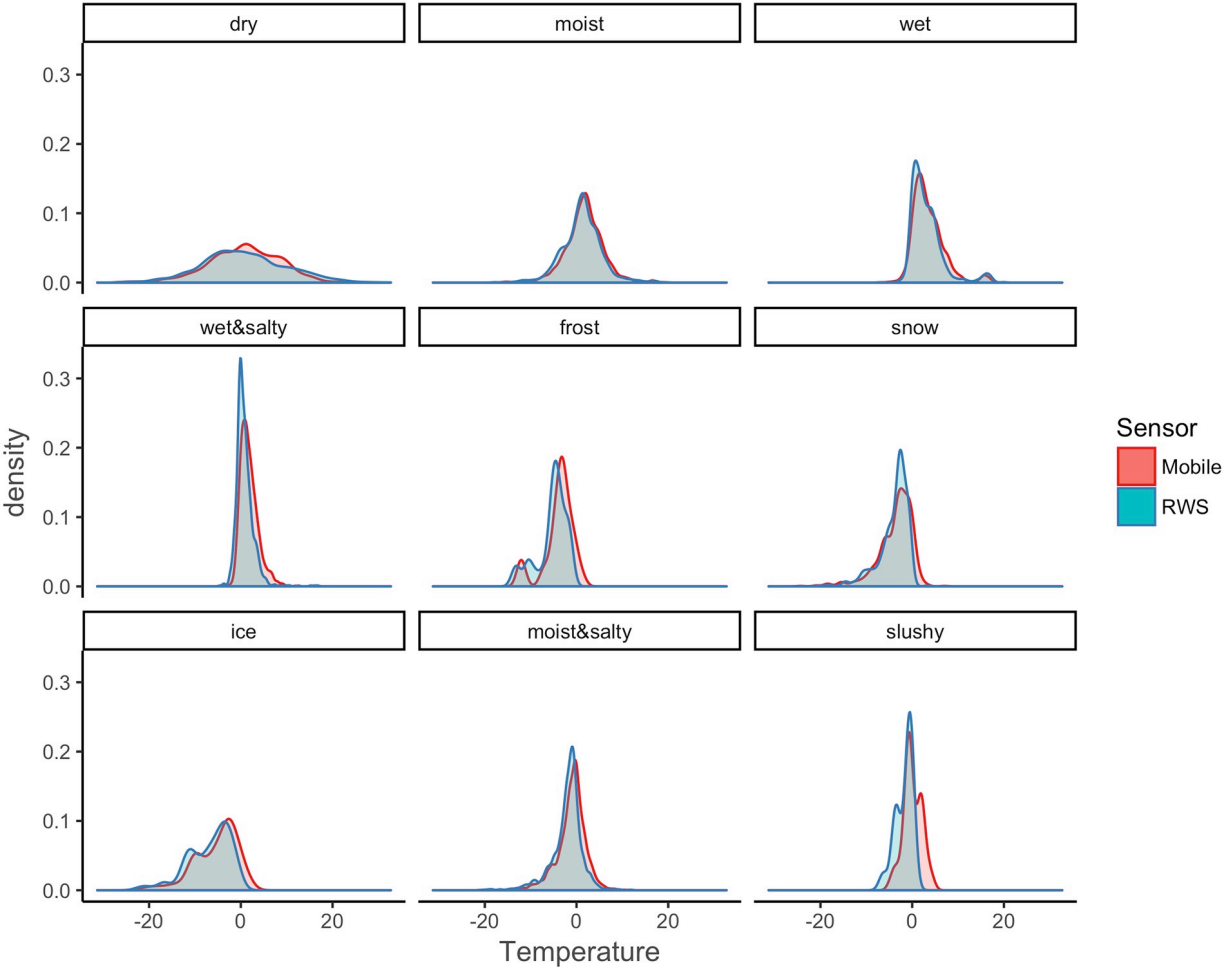
Surface temperature observations for each RWS road status.

**Fig 3:**
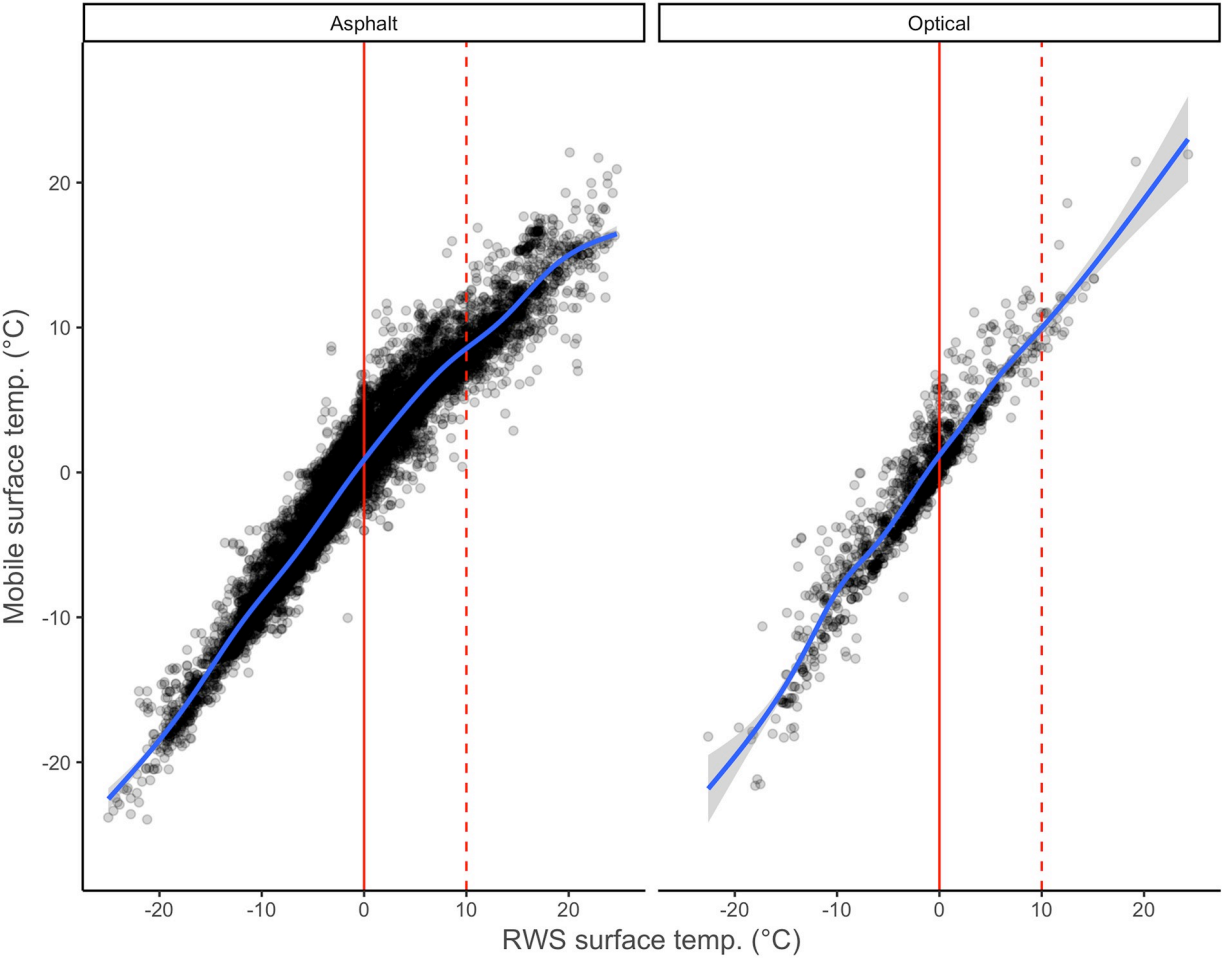
Scatterplot of RWS and mobile surface temperature observations. Nonparametric (LOESS) prediction line is shown in blue, the 0°C level in solid red, and 10°C in dashed red.

Surface temperature densities for each RWS road status are depicted in Fig 2. They are almost identical, which indicates that the mobile and the RWS observations largely agree. However, the mobile observations appear slightly higher than the RWS observations. A scatterplot (see Fig 3) between the mobile and the RWS surface temperature observations further reveals that while the observations largely agree, following a linear relationship, between 0°C and 10°C the LOESS prediction line for the asphalt embedded sensor takes a sudden downward turn.

**Fig 4:**
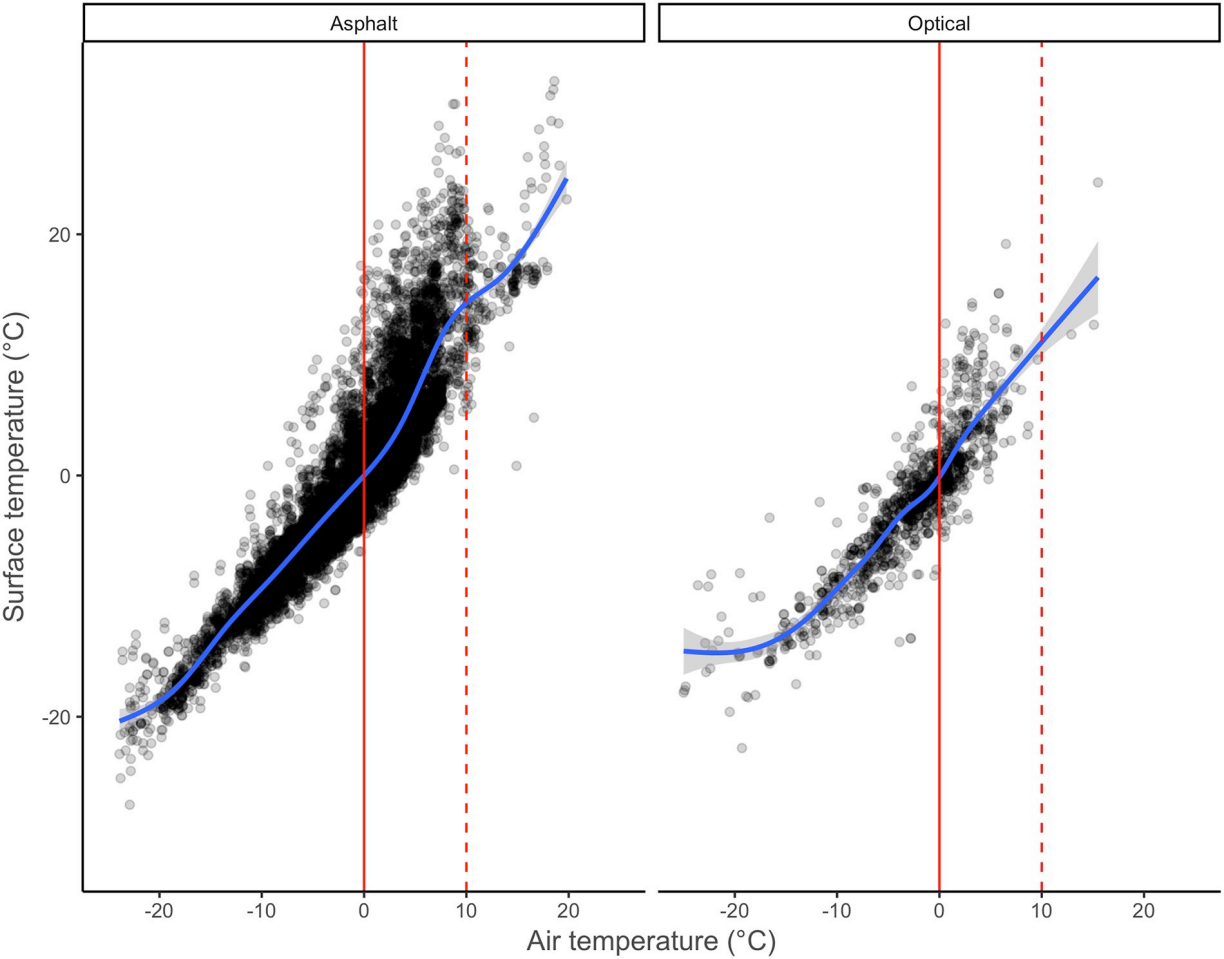
Scatterplot of RWS surface and air temperature observations. Nonparametric (LOESS) prediction line is shown in blue, the 0°C level in solid red, and 10°C in dashed red.

Fig 4 shows that the correlation between RWS air and surface temperatures is strong. Again, there is a change in slope between 0°C and 10°C for the asphalt embedded sensor.

**Fig 5:**
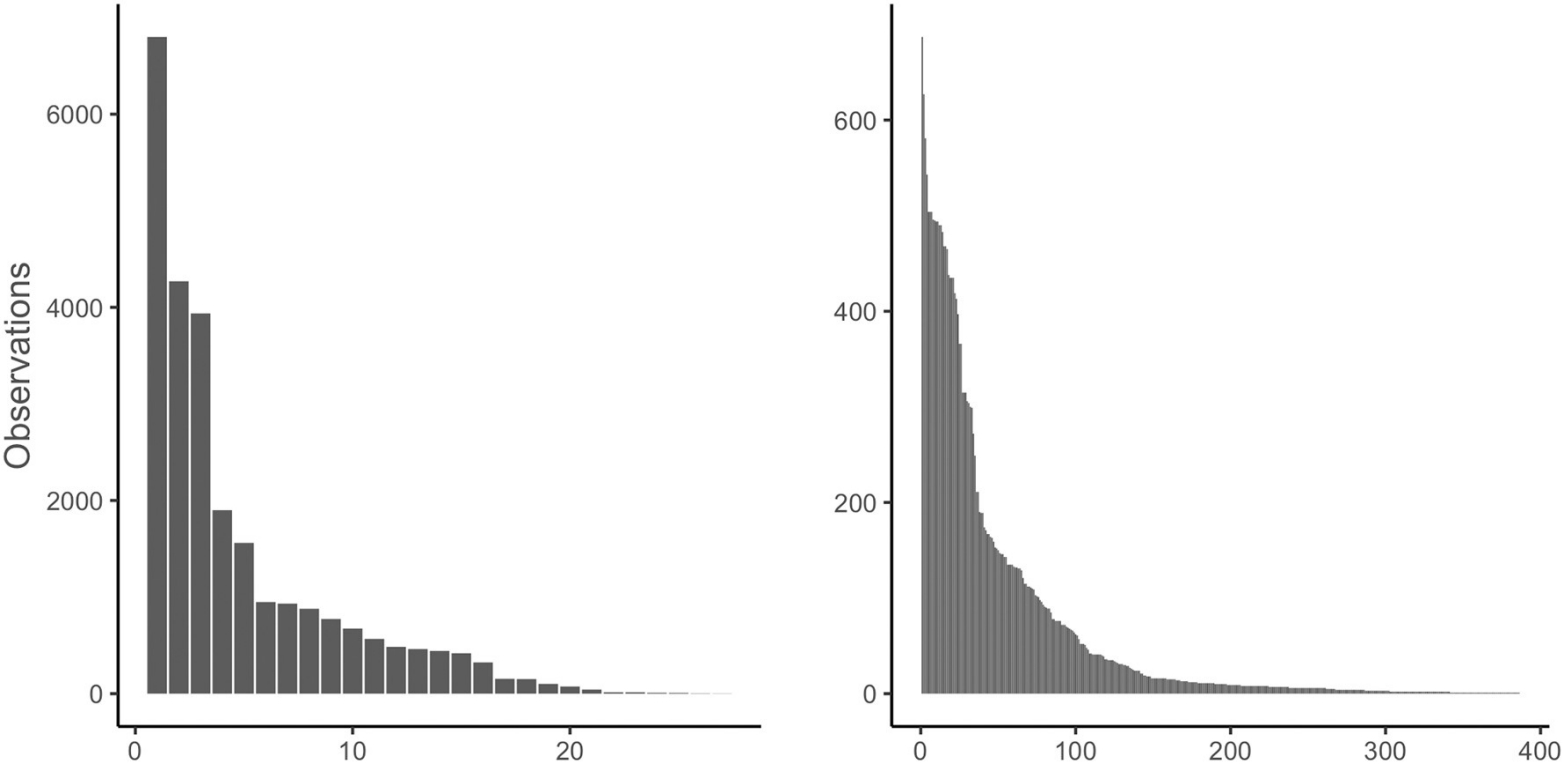
Histograms of observations on RWS (left) and mobile (right) sensors.

The number of observations on individual RWS and mobile sensors follow similar patterns (Fig 5). There are few sensors with hundreds or even thousands of observations, and a long tail with just a few.

**Fig 6:**
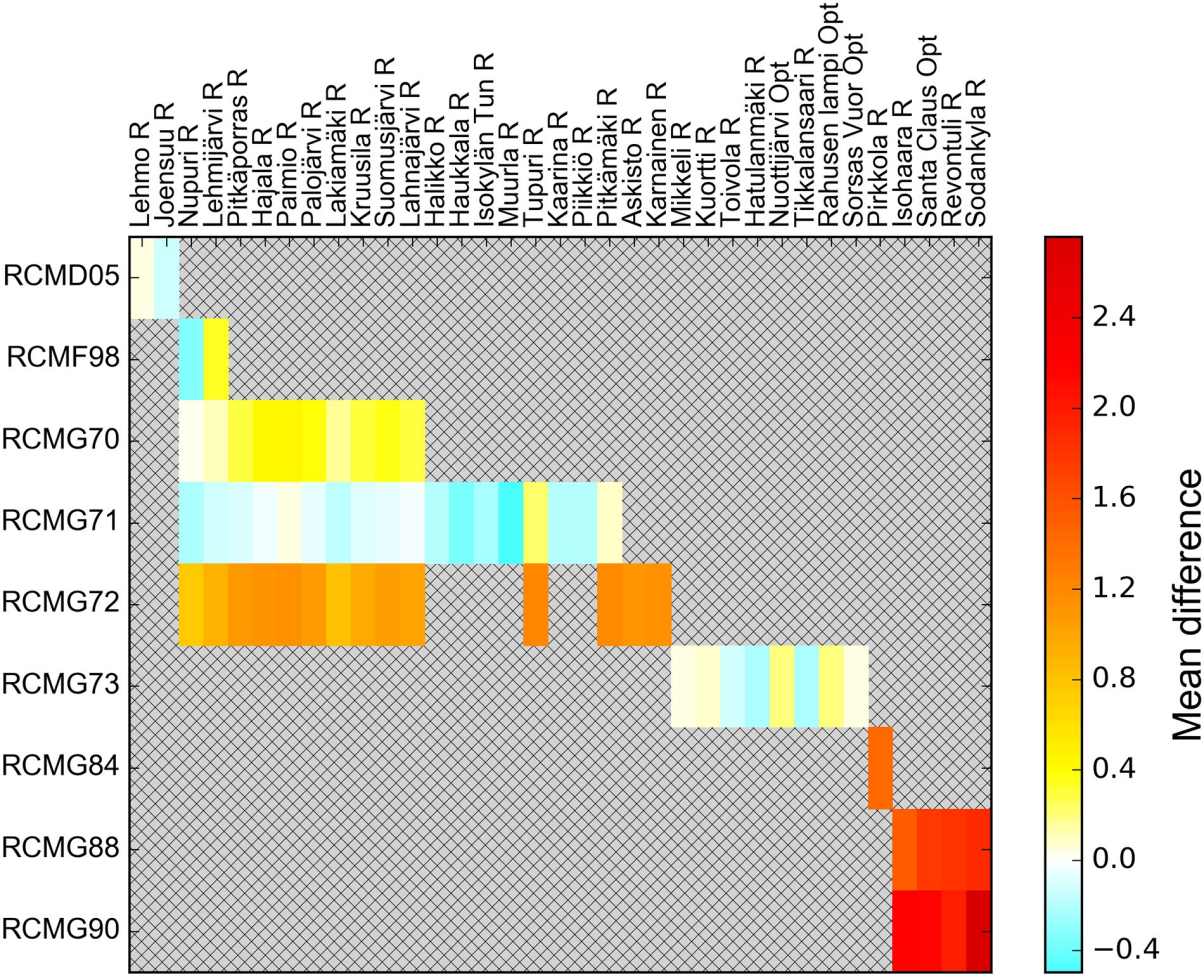
Average difference between each mobile–RWS observation pair. Stations marked with “R” measure surface temperature with a Vaisala DRS511, embedded in the asphalt, while stations marked with “Opt” have optical Vaisala DST111 instruments. When the RWS and/or the mobile observation is above 8°C, the pair is discarded. Further, each mobile–RWS pair have at least 40 measurements.

Average differences between individual RWS and mobile sensors vary largely (Fig 6). The differences seem to depend more on the mobile sensor device than the RWS. One mobile device tends to diverge from the RWSs roughly the same amount, regardless of the RWS. This suggests that the vehicle and the installation of the mobile sensor device affect the observations significantly.

The number of distinct sensors per year is listed in Table 2. The number of mobile sensor installations are steadily increasing, and their reach of RWSs consequently rising. (Note that the last observation was taken at 2017-04-30).

**Table 2:** Number of distinct sensors per year.

	2015	2016	2017
Mobile	14	17	22
RWS	199	324	295

Categorizing the observations of the full data set by both the mobile and the RWS weather status observations, the data are distributed as depicted in Table 3. While the diagonal of Table 3 dominates, there are many observations where the mobile and the RWS weather states differ on a fundamental level. In part, this is explained by the way our observations were collected, as the driving conditions can be different within the at most 50m distance between the mobile and the RWS sensor. Still, it is clear that the devices interpret their surrounding environments in different ways.

**Table 3:** Number of observations by mobile and RWS road status.

Mobile	RWS								
	Dry	Moist	Wet	Wet&Salty	Frost	Snow	Ice	Moist&Salty	Slushy
Dry	4097	497	63	45	0	16	205	565	2
Moist	927	841	187	224	2	21	123	1773	2
Wet	344	1401	1363	1381	1	26	34	3113	5
Slush	10	30	39	78	0	62	31	212	12
Ice	379	160	28	42	34	648	450	582	13
Snow/frost	14	3	0	0	1	133	47	11	0

### Statistical models

A linear regression model estimates a linear and additive relationship between independent variables and a response. Such a model may contain a varying number of fixed effects, estimating the *ceterus paribus* relationship between individual independent variables and the response. However, there may be only one random effect: the noise term.

In contrast, a linear mixed effects model [19, 20] may contain several random effects. If an independent variable is related to a random effect instead of a fixed one, the model no longer estimates a coefficient for inferring the relationship between the variable and the response. Instead, the effect is assumed to follow a zero-mean Gaussian distribution.

Linear mixed models are computationally light and can be used for both statistical inference and prediction. In this study, mixed models are first used inferentially, to find out how circumstantial variables as well as the weather conditions, as measured by both the mobile sensors and the RWSs, affect the quality of the road surface measurements. Second, linear mixed models are used predictively to calibrate the mobile sensors to agree with the RWSs. A full list of variables is shown in Table 1.

Simple linear regression models provide a baseline for comparison of the inferential and the predictive cases. The baseline inference models A0 and A00 are defined as:

$$T_{s,\text{Mob}}=X_{\text{RWS},\;\text{id}}\beta +\varepsilon ,$$ (1)

where *T*_*s*,Mob_ is mobile surface temperature, *X*_RWS,id_ is a (*n* × *p*) design matrix consisting of the intercept, the RWS surface temperature observations, and (for A00) dummy variables for the sensor id codes, controlling for the individual sensor factory calibration levels. Factory calibration level here refers to a device’s reading when the surface temperature is actually 0°C. This could be affected by the internal settings of the device as well as the installation on the vehicle (by way of e.g. proximity to exhaust fumes). *ε* is a vector of random noise with $\varepsilon ∼N(0,I\sigma_{e}^{2})$. A0 and A00 are used to provide a reference point for assessing the quality of the mixed models A1–A4 that were defined as

$$T_{s,\text{Mob}}=X_{\text{RWS}}\beta +Z_{\text{id}}b+\varepsilon ,$$ (2)

where *X*_RWS_ is a (*n* × *p*) design matrix consisting of the intercept, the RWS weather observations, and some circumstantial data, while *Z*_id_ is a design matrix for the dummy variables of the mobile and RWS sensor identification codes. Finally, *ε* is a vector of random noise with $\varepsilon ∼N(0,I\sigma_{e}^{2})$.

The *β* parameters are considered fixed effect coefficients. The parameters *b* = (*b*_1_, *b*_2_) are considered random effect coefficients and correspond to the individual factory calibration levels of the devices. *b*_1_ refers to mobile sensors and *b*_2_ to RWS sensors, with $b_{1}∼N\left(0,I\sigma_{1}^{2}\right)$ and $b_{2}∼N\left(0,I\sigma_{2}^{2}\right)$. Thus, the model assumes the individual factory calibration levels of the sensor devices are normally distributed with zero means but separate variances for the two device types.

The aim of the predictive modelling is to predict the RWS surface temperature using only mobile observations. We have a baseline linear model P0:

$$T_{s,\text{RWS}}=X_{\text{Mob}}\beta +\varepsilon ,$$ (3)

where *T*_*s*,RWS_ is RWS surface temperature, *X*_Mob_ is a (*n* × 2) design matrix consisting of the intercept and the mobile surface temperature observations, and *ε* is a vector of random noise with $\varepsilon ∼N(0,I\sigma_{e}^{2})$. P0 is used to provide a reference point for assessing the prediction quality of the mixed models P1–P3, defined as:

$$T_{s,\text{RWS}}=X_{\text{Mob}}\beta +Z_{\text{id}}b+\varepsilon ,$$ (4)

where *X*_Mob_ is a (*n* × *p*) design matrix consisting of the mobile road weather observations, *Z*_id_ is a design matrix for the two dummy id code variables, and *ε* is a vector of random noise with $\varepsilon ∼N(0,I\sigma_{e}^{2})$. Again, the *β* parameters are considered fixed, while the *b* = (*b*_2_, *b*_2_) parameters are normally distributed.

### Model quality

This study uses the conditional Akaike Information Criterion (conditional AIC) [21] to compare the quality between models. Further, mixed effect models have the following assumptions: (1) the independent variables (i.e. the columns in the model matrices X and Z) should not be correlated, (2) the residuals should have a constant variance, (3) the residuals should be uncorrelated, and (4) normally distributed with (5) zero means, and finally, the random effects should be (6) normally distributed. The inferential model was tested for each of these assumptions.

The Variance Inflation Factors (VIFs) [22] for the inference model variables {*RWS road state, >4°C, RWS surface temp., RWS water deposits, RWS ice deposits, RWS Snow deposits, Year, Season, Distance of sensors*} are, respective, {4.4, 1.8, 2.2, 1.2, 2.0, 1.4, 1.3, 1.8, 1.0}. These are well below 10, considered as severe multicollinearity [22], and thus in agreement of Assumption 1.

Including the RWS air temperature variable in the model raises the RWS surface temperature VIF score above 10. This is in line with the observed high correlation in Fig 4. Consequently, RWS air temperature is excluded from the models.

S1 Fig shows the residual vs. fitted plot of the associative model A4. The plot indicates the residuals are with constant variance (Assumption 2) and with zero means (Assumption 5). Further, no autocorrelation is visible in the plot, fulfilling Assumption 3.

The Quantile-Quantile plots in S2, S3 and S4 Figs indicate validity of assumption that the residuals and random effects follow the normal distribution (assumptions 4, 6) moderately well. While the plots indicate the residual and the RWS calibration level distributions have somewhat heavier than Gaussian tails, and the mobile sensor calibration level distribution is more mesocurtic, the bulk of the standardized quantiles fall on the diagonal. Further, all distributions appear symmetrical, suggesting the divergence in the tails, while adding variance, does not add bias in our estimates.

For the predictive models, the main point of interest is the prediction accuracy. Accordingly, we measure the prediction accuracy of each model by the cross-validated mean squared error (MSE). The cross-validated MSE is calculated as the average of the MSEs of 10 unstratified folds. Further, the coefficient estimates for the predictive models are the averages of those of each fold.

## Results and discussion

### Inference models

**Fig 7:**
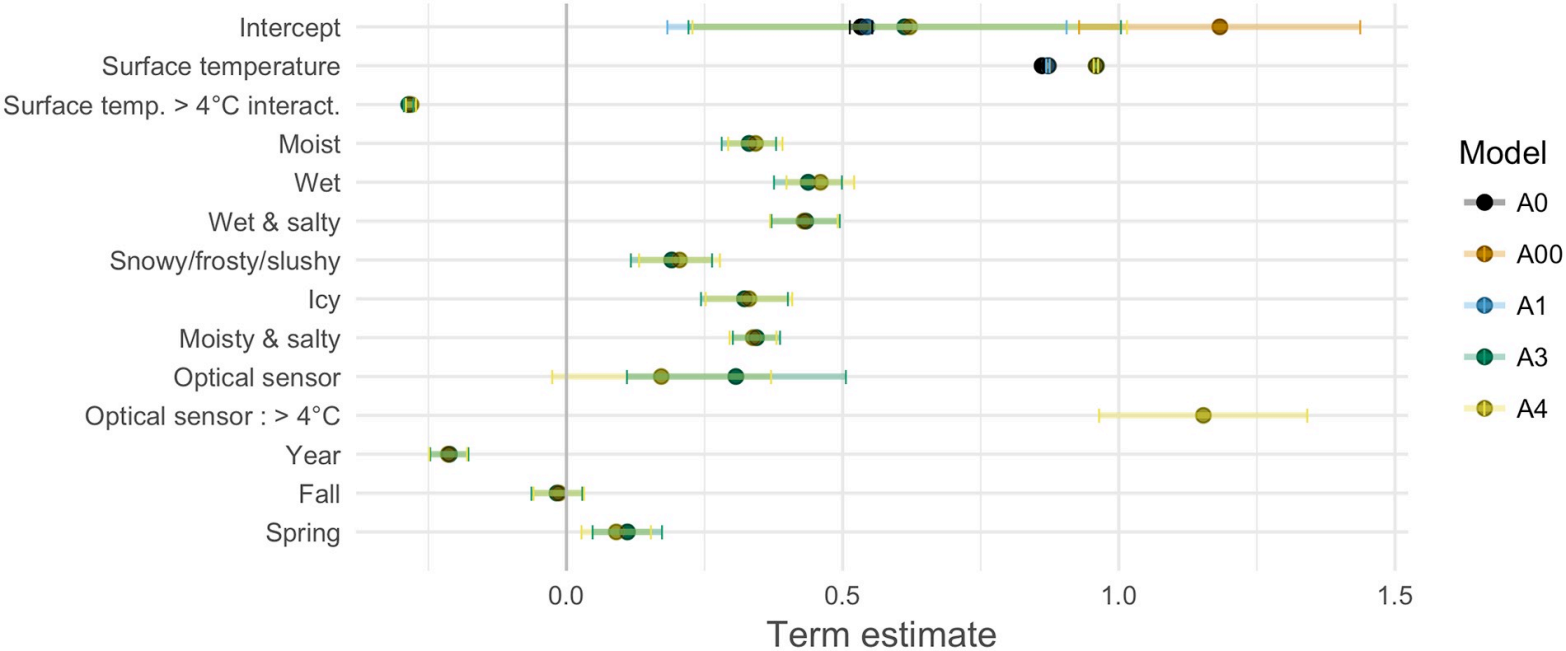
Estimates and 95% confidence intervals of fixed coefficients for the inference models. Model A2 excluded due to term instability.

Coefficient estimates and model quality indicators for the inference models are listed in Table 4, with the inference model coefficients further detailed in Fig 7.

**Table 4:** Estimates of fixed effect coefficients and quality metrics for the inference models.

Term	A0 (linear)	A00 (linear)	A1 (mixed)	A2 (mixed)	A3 (mixed)	A4 (mixed)
Intercept	0.53 (0.01)	1.18 (0.13)	0.54 (0.18)	1.42 (0.40)	0.61 (0.20)	0.62 (0.20)
Sensor calibr. levels	no	fixed	random	random	random	random
RWS surface temp.	0.86 (0.00)	0.87 (0.00)	0.87 (0.00)	1.05 (0.01)	0.96 (0.00)	0.96 (0.00)
> 4°C interact.				-0.26 (0.04)	-0.29 (0.01)	-0.28 (0.01)
Moist				0.15 (0.11)	0.33 (0.03)	0.34 (0.03)
Wet				0.17 (0.14)	0.44 (0.03)	0.46 (0.03)
Wet & Salty					0.43 (0.03)	0.43 (0.03)
Snowy/frosty/slushy				0.10 (0.14)	0.19 (0.04)	0.21 (0.04)
Icy				0.13 (0.18)	0.32 (0.04)	0.33 (0.04)
Moisty & salty					0.34 (0.02)	0.34 (0.02)
Optical sensor					0.31 (0.10)	0.17 (0.10)
>4°C interact.						1.15 (0.10)
Water deposits				-0.64 (0.34)		
Ice deposits				1.22 (0.53)		
Snow deposits				-0.26 (0.31)		
Year				0.33 (0.09)	-0.21 (0.02)	-0.21 (0.02)
Fall				-0.06 (0.16)	-0.02 (0.02)	-0.01 (0.02)
Spring				0.01 (0.12)	0.11 (0.03)§	0.09 (0.03)
conditional AIC	73138	65210	65377		59134	59004
R^2^	0.927	0.951	0.953	0.954	0.965	0.966
N	20278	20278	20278	1283	20278	20278

For estimates, the standard error is provided in parentheses.

A0 is the baseline inference model, with just the intercept and the slope adjustment to the RWS surface temperature value. A00 still has only fixed effects, but includes additionally the individual sensor calibration levels as categorical variables. A1 assumes the underlying distribution of the calibration levels is Gaussian and considers them as random effects. A2–A4 include various other variables into the model.

Model quality considerations (see section Model quality) show a slight deviation from the Gaussian assumption for the residuals, which potentially causes noise in the estimates. This is possibly one cause of the relatively large standard error seen in the model intercepts (see Table 4 and Fig 7). Apart from the underlying error term distribution, the large intercept standard error may be caused by the spatial and temporal difference between the mobile and the stationary observations.

The estimates by the baseline A0 model are well in agreement with those of the mixed A1, A3 and A4 models. On the other hand, model A00 intercept’s value is markedly higher, reaching 1.2°C. The difference between A00 and A1, a corresponding mixed model, is especially striking, with A1 intercept at 0.54°C. The reason behind the difference may be the uneven distributions of observations by the different sensors. As seen in Figs 5 and 6, the distribution of observations on individual sensors is very uneven, and the factory calibration levels appear to vary greatly. The simple linear model A00 is not robust enough to cope with sensors whose outlying factory calibration levels skew the intercept with even a small number of observations, which is a further justification for the use of the linear mixed models.

Interestingly, the A00 intercept exactly agrees with the 1.2°C reported by Malmivuo [5]. Malmivuo’s method investigated the medians of the mobile Teconer RTS411 and the RWS observations. The model itself is comparable with our A0, albeit somewhat more robust to outliers. The difference between the results may be due to Malmivuo’s study only utilizing the measurements of one mobile sensor, whose factory calibration level, as seen in Fig 6, can be off the actual.

Models A1, A3 and A4 largely agree on their coefficient estimates, with the small confidence intervals indicating high reliability in the estimates. Ultimately, the conditional AIC model quality criteria chose model A4 as the one best describing the data.

Due to the relatively low number of observations, the mixed inference model A2 is somewhat unstable, with large confidence intervals for many coefficient estimates. However, the model suggests that ice deposits on the road tend to cause the mobile sensor device to have higher than average surface temperature readings or, conversely, the RWS observations to fall under the average. This may be caused by the asphalt embedded RWS sensors (Vaisala DRS511), whose temperature readings can vary a lot from an optical one measuring the surface of the ice.

Relying on the A4 model, in dry conditions, at 0°C surface temperature, the mobile observations are on average 0.62 degrees higher than those of the RWS. However, there is considerable variation in the estimate, with a 95% confidence interval spanning [0.23°C, 1.0°C]. Further, different driving conditions have quite a strong effect on the difference. For example mobile observations are further 0.46 degrees higher in wet conditions, resulting over one degree above RWS in total.

Surface temperature coefficient is very close to 1 as expected. This indicates that mobile and RWS observations indeed almost agree when surface temperature is under 4°C and after level adjustments provided by the different driving conditions are taken into account. A marked discrepancy appears between the observations above 4°C, with the mobile sensor reporting observations consistently and increasingly lower than the RWS. This observation is in line with Fig 3 that shows a distinct turn in the prediction curve at around 4°C for the asphalt embedded sensors, comprising a large majority of observations.

RWS sensor type does not have an effect on the measurements under 4°C. However, model A4 indicates that the optical DST111 RWS sensor gives as much as 1.2°C lower readings than the asphalt embedded DRS511 above 4°C. This can be caused by the asphalt embedded DRS511 heating up too much in direct sunlight during sunny autumn and spring days. The observation is again in line with Fig 3, where the optical sensor has a much straighter prediction line compared to that of the one embedded in the asphalt.

The season of observation has little influence on the observations. However, there is a significant yearly trend in the measurements. Teconer observations seem to drop by ca. 0.2°C in relation to RWS observations year-by-year. This may be caused by wear-and-tear in the mobile sensors, e.g. by dirt or scratches accumulating on the optics. Another potential cause is adjustments in the mobile sensor installations that were not included in the data available for this study. Either way, a continuous degradation calls for recurrent sensor calibration.

### Prediction models

Coefficient estimates and model quality indicators for the prediction models are listed in Table 5. The mean squared error (MSE) of the predictive models P0–P3 indicate that the RWS surface temperatures predicted by model P1 follow the observed ones most closely.

**Table 5:** Estimates of fixed effects coefficients and performance metrics for the prediction models.

Term	Obs.	P0 (linear)	P1 (mixed)	P2 (mixed)	P3 (mixed)
Intercept		-0.62			
Teconer surface temp.		1.075	1.132	1.089	1.083
Moist interact.			-0.112		
Wet interact.			-0.093		
Snowy/frosty interact.			-0.233		
Icy interact.			-0.159		
Slushy interact.			-0.174		
Dry			-0.027	0.004	
Moist			-0.793	-0.747	
Wet			-0.958	-1.047	
Snowy/frosty			-1.001	-0.601	
Icy			-0.996	-0.508	
Slushy			-1.014	-0.946	
MSE	3.252	2.747	1.923	2.051	2.212
N	20305	20305	20305	20305	20305
folds		10	10	10	10

Estimates and metrics are both calculated by averaging the results of all folds in a 10-fold cross-validation set-up.

Unmodified mobile observations had an MSE of 3.3. Adjusting the mobile observation with the average difference between the observations (-0.62, taken from the intercept-only regression model P0) would slightly improve the result, reaching MSE 2.7. However, the use of linear mixed models P1–P3 improve the result markedly. P1 MSE is 1.9, increasing the accuracy of the unmodified mobile observation by more than 40%.

Model P1 includes interaction terms between each road status and the RWS surface temperature, in addition to level adjustments. This changes the slope of the linear relationship between RWS and mobile observations. The following linear formula can thus be applied to calibrate the mobile Teconer RTS411 observations to better agree with the RWS:

$$T_{s,\text{Adj}}=\beta_{0}+\beta_{1}\times T_{s_{,}\text{Mob}},$$ (5)

where *T*_*s*,Adj_ is the adjusted mobile surface temperature, *β*_0_ is the level coefficient and *β*_1_ is the slope coefficient. Table 6 lists the level and slope coefficients for adjusting the mobile surface temperature in the observed road status, extracted and rounded from model P1 in Table 5.

**Table 6:** Linear coefficients for adjusting the mobile Teconer RTS411 road surface temperature observations.

Road status	*β*_0_	*β*_1_
Dry	-0.03	1.13
Moist	-0.79	1.02
Wet	-0.96	1.04
Snow/frost	-1.00	0.91
Ice	-0.97	0.97
Slush	-1.01	0.96

Look up the Teconer RCM411 road status observation and select the coefficients accordingly.

To summarize, the inference models captured the effect of the environmental conditions on the mobile sensor observations. Dry, moist, wet, snowy, icy and slushy driving conditions all made subtle changes to the mobile observations, while a small but significant degradation was evident from a yearly trend. Further, a linear calibration, whose coefficients were estimated by fitting a linear mixed model, reduced the mobile sensor error by ca. 40%.

## Conclusion

Mobile, vehicle-installed sensors and RWS networks can together provide denser and higher quality information of challenging driving conditions than either alone. First, this study analyzed the observations of mobile (Teconer RCM411, RTS411) and RWS (Vaisala DRS511, DST111, DSC111) road weather sensors and identified conditions and factors that affect how their measurements differ from each other. Second, this study presented a novel calibration method for the mobile sensors. A straightforward linear Eq (5) adjusted the mobile road surface temperature observations to be consistent with the stationary ones, halving the MSE between the mobile and the RWS observations.

The analysis and the calibration were based on rendezvous model sensor fusion, which considers spatio-temporally co-located observations of mobile and RWS sensors. Further, both analysis and calibration used linear mixed models to compare the mobile and RWS observations, taking into account the prevailing driving conditions as well as other environmental factors. The analysis revealed the following:

1. In dry conditions, at 0°C surface temperature, with no water, ice, or snow deposits on the road, the mobile observations are on average 0.62 degrees higher than those of the RWS.
2. Different driving conditions, indicated by the RWS road status variable, have a significant effect on the difference. For example, mobile observations are a further 0.46°C higher than RWS observations in wet conditions, totalling over 1°C above RWS observations.
3. Model A4 indicates that the optical Vaisala DST111 RWS sensor gives on average 1.2°C lower readings than the asphalt embedded Vaisala DRS511 in surface temperatures above 4°C.
4. Mobile Teconer RTS411 sensor observations fall by ca. 0.2°C year-by-year, justifying recurrent recalibrations.

Linear mixed models assume the general noise term is Gaussian. Model diagnostics revealed that while moderately well adhering to this assumption, the residuals of the inference models suggest the models could be further improved by replacing the Gaussian noise term distribution with one thicker tailed. For future work, we suggest embracing the Bayesian inference framework, with the noise term following e.g. Student’s t distribution. Such an approach may reduce the relatively large standard error of the reported intercept estimates of the inference models. Further, the MSE’s of the predictive models may be further lowered.

The sensor calibration method proposed in this study, based on sensor fusion and linear mixed effect models, is computationally very light. The linear calibration function can be embedded directly in the sensors. Further, the calibration method can be further utilized in other application areas such as air quality, temperature, and usage monitoring in smart building scenarios.

## Supporting information

S1 Fig Residual plot of the inference model. (TIF)

S2 Fig Q-Q plot of a sample of 100 residuals. (TIF)

S3 Fig Q-Q plot of the RWS sensor calibration level random effect. (TIF)

S4 Fig Q-Q plot of the mobile sensor calibration level random effect. (TIF)
