# Supplementary figures and images for: Mobile road weather sensor calibration by sensor fusion and linear mixed models
Source: PLoS One. 2019 Feb 7;14(2):e0211702. doi: 10.1371/journal.pone.0211702 (PMC6366776; doi:10.1371/journal.pone.0211702)

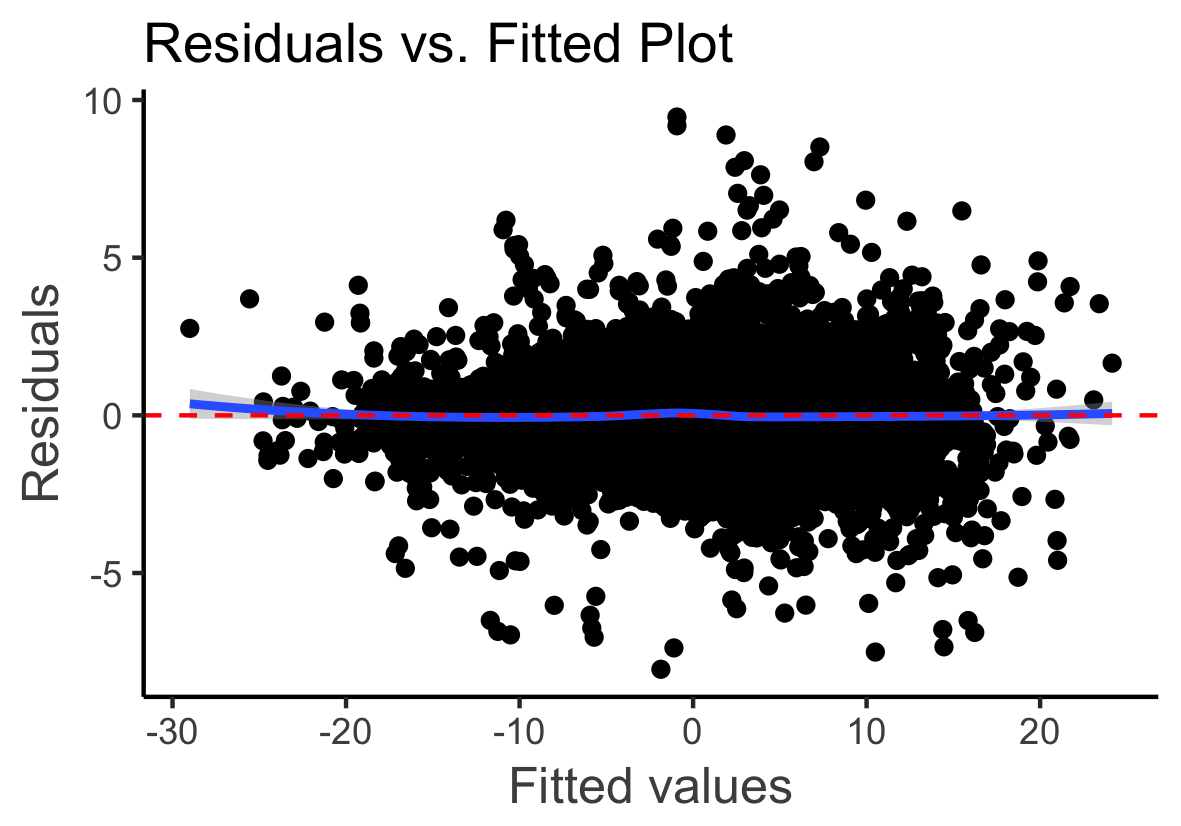

Supplement: S1 Fig — (TIF) [file pone.0211702.s001.tif]

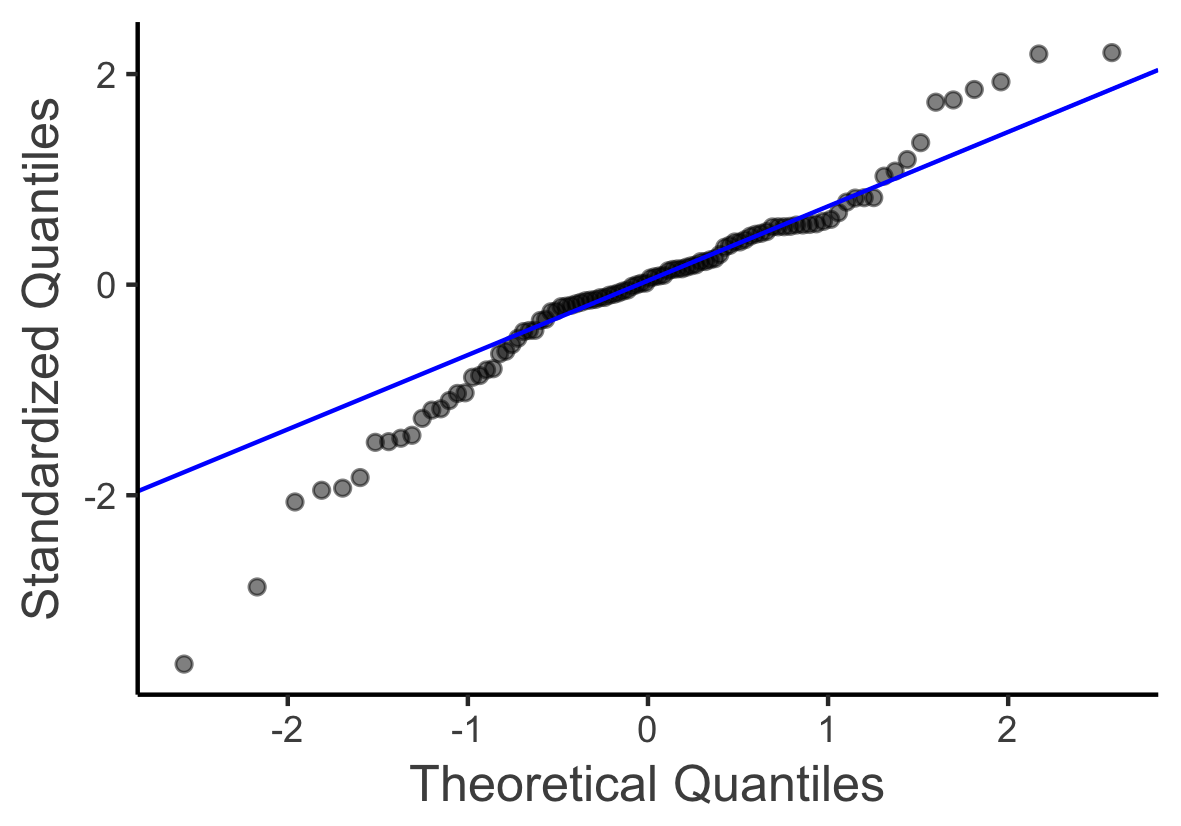

Supplement: S2 Fig — (TIF) [file pone.0211702.s002.tif]

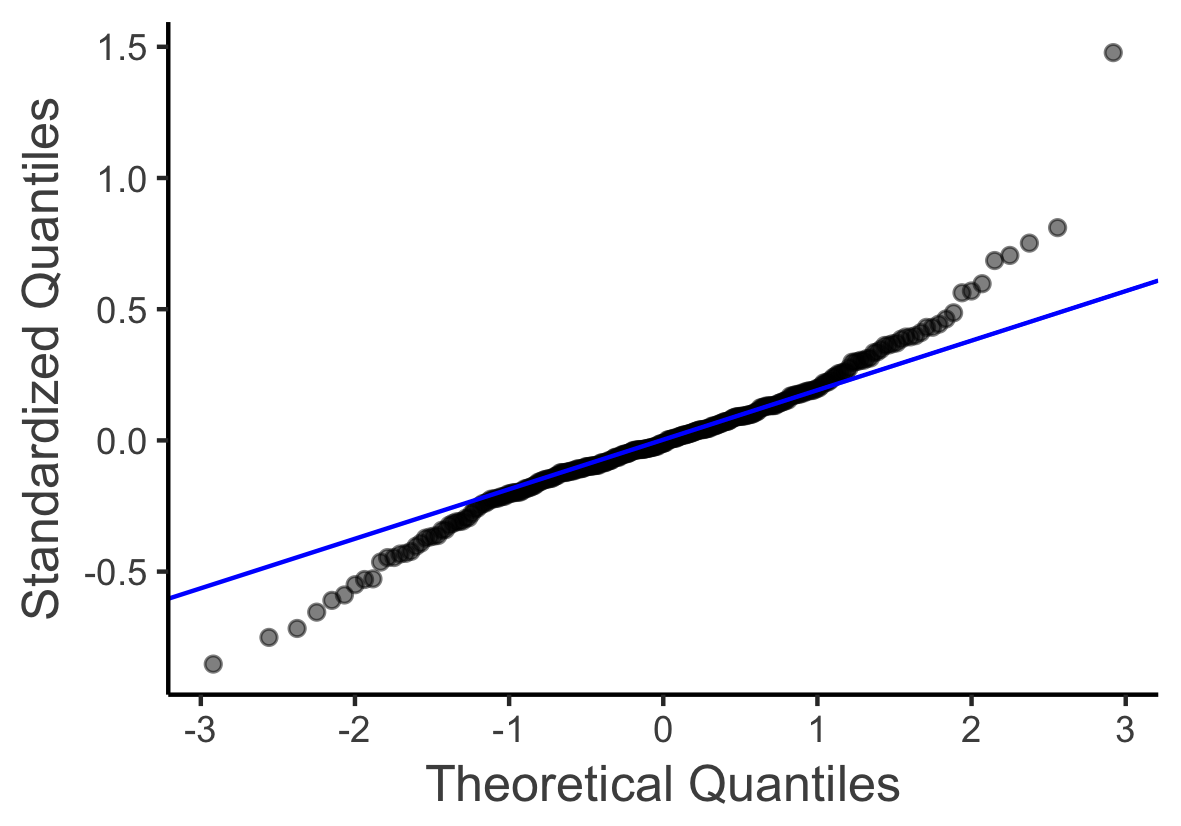

Supplement: S3 Fig — (TIF) [file pone.0211702.s003.tif]

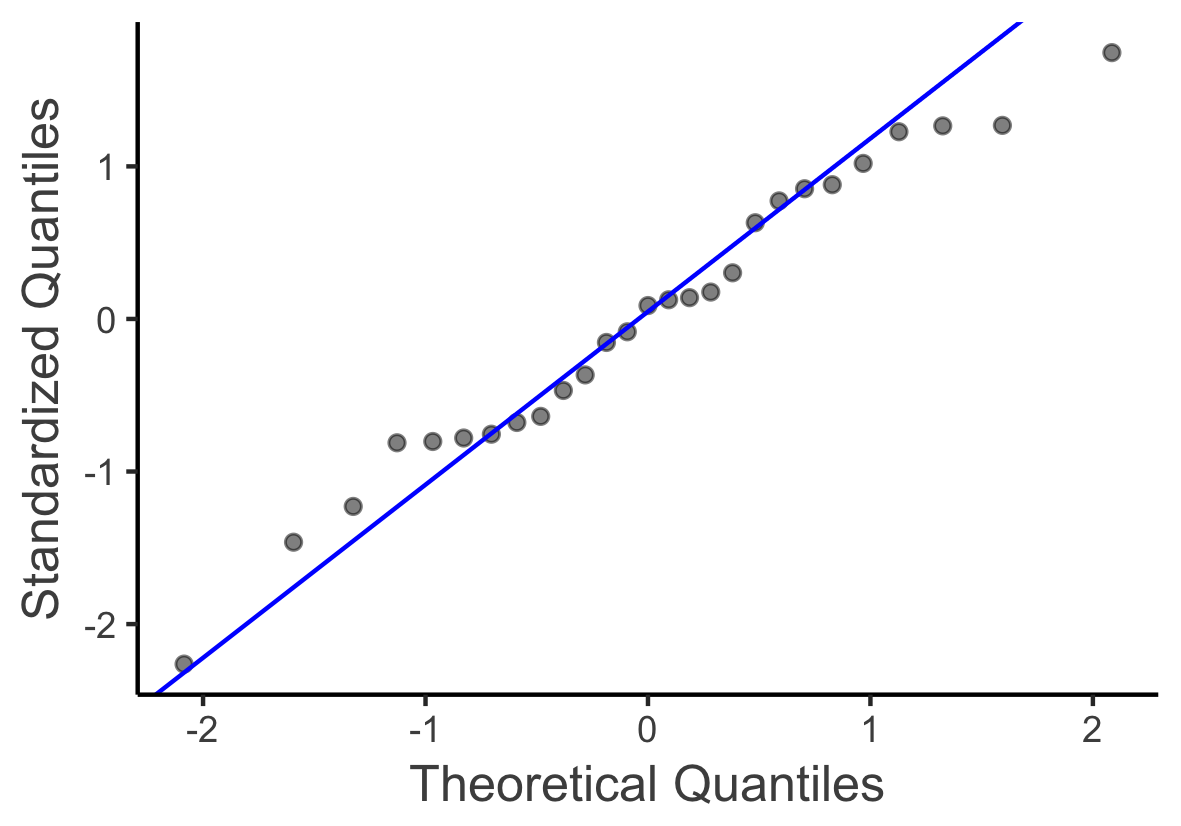

Supplement: S4 Fig — (TIF) [file pone.0211702.s004.tif]
